# Orexin facilitates the peripheral chemoreflex in the active phase via corticotropin-releasing hormone neurons that project to the nucleus of the solitary tract

**DOI:** 10.1152/function.084.2025

**Published:** 2025-12-08

**Authors:** Ruwaida Ben Musa, Fateme Khodadadi-Mericle, David D. Kline, Eileen M. Hasser, Kevin J. Cummings

**Affiliations:** Department of Pathobiology and Integrative Biomedical Sciences, College of Veterinary Medicine, Dalton Cardiovascular Research Center, University of Missouri, Columbia, Missouri, United States

**Keywords:** chemoreflex, corticotropin releasing hormone, hypoxia, nucleus of the solitary tract, orexin

## Abstract

Projections from the paraventricular nucleus (PVN) of the hypothalamus to the nucleus of the solitary tract (nTS) facilitate the peripheral chemoreflex. A significant proportion of this projection is composed of corticotropin-releasing hormone (CRH) neurons. Orexin neurons in the perifornical hypothalamus augment the peripheral chemoreflex, project to the PVN, and facilitate the hypoxia-induced activation of nTS-projecting CRH neurons. We hypothesized that nTS-projecting CRH neurons are necessary for the full reflex, and that orexin facilitates the reflex via the CRH-nTS pathway. We chemogenetically silenced or activated nTS-projecting CRH neurons during normoxia and acute hypoxia. For each rat, reflex strength was tested in both inactive and active phases as the activity of orexin neurons is phase dependent. Testing was done following vehicle, Compound 21 (1 mg/kg) to activate Gi- or Gq-DREADDs, and after systemic Ox1R blockade (SB-334867; 1 mg/kg). We performed immunohistochemistry to assess how chemogenetic manipulation of nTS-projecting CRH neurons influenced their activation by hypoxia (via cFos). Activating the CRH-nTS pathway had no effect on the chemoreflex in either phase. Silencing the pathway in the active phase, but not inactive phase, reduced the strength of the reflex by ∼50% and prevented further inhibition by Ox1R blockade, suggesting orexin acts via Ox1R on CRH neurons. Pathway silencing reduced the proportion of nTS-projecting CRH neurons activated by hypoxia, consistent with the effects of pathway silencing on the reflex. These data suggest that orexin augments the peripheral chemoreflex in the active phase via the CRH-nTS pathway.

## Graphical abstract

**Figure:**
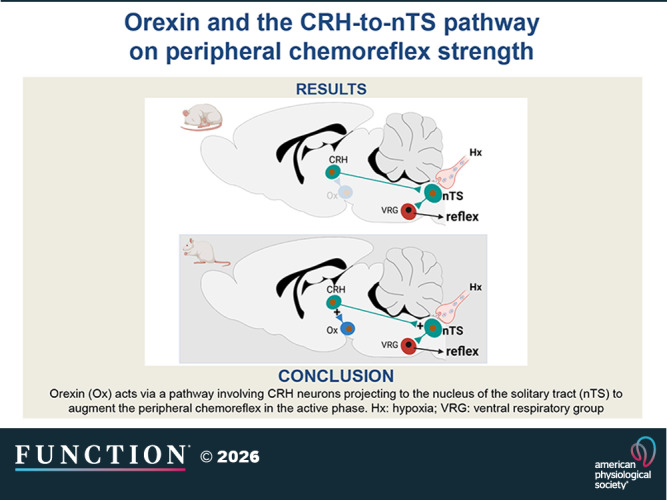

## INTRODUCTION

The peripheral chemoreflex, originating at the carotid body chemoreceptors, elicits the adaptive respiratory and autonomic responses to hypoxia and the excessive, maladaptive increase in sympathetic nervous system activity associated with a variety of cardiorespiratory disease states (1). Canonical brainstem nuclei mediating the reflex include the nucleus of the solitary tract (nTS), the site receiving and integrating chemoreceptor afferent information. Projections from the nTS subsequently activate premotor sympathetic and respiratory neurons in the ventrolateral medulla to ultimately initiate increases in sympathetic nervous system activity and breathing (2). The strength of the reflex motor responses also relies on forebrain nuclei. For example, the paraventricular nucleus (PVN) of the hypothalamus is necessary for autonomic and neuroendocrine responses to a variety of physiological stressors (3, 4), including the peripheral chemoreflex-mediated cardiorespiratory responses to hypoxia. Indeed, the PVN is activated by hypoxia (5–7), and experimentally compromising PVN activity—historically this was done using electrolytic lesions or direct pharmacological inhibition—reduces the strength of the reflex (8, 9). More recently, our group has shown that chemogenetically silencing the PVN globally and specifically the entire projection from PVN to nTS also decreases the magnitude of cardiovascular and phrenic nerve responses to peripheral chemoreceptor stimulation (5–9).

PVN neurons can alter cardiorespiratory motor activity via projections to multiple CNS regions. Projections to the ventrolateral medulla (VLM) and spinal cord can alter reflex responses to a variety of acute and chronic physiological stressors (3). Hypoxia, however, is an exception that does not appear to activate PVN-VLM circuits (10). Instead, hypoxia recruits a heavy projection from the PVN to the nTS (5) that supports the cardiorespiratory responses elicited by activation of the peripheral chemoreflex (6, 7). The PVN-to-nTS projection contains multiple neuronal phenotypes; however, the component of the projection primarily activated by hypoxia consists of neurons immunoreactive for corticotropin-releasing hormone (CRH) (5, 11). This observation suggests that the bulk of the support for the peripheral chemoreflex responses provided by the PVN-nTS projection comes from CRH neurons.

Orexin neurons in the perifornical hypothalamus contribute to cardiorespiratory regulation, including the sympathetic and ventilatory responses to CO_2_ (12). Recently, we showed that orexin also contributes to the peripheral chemoreflex-mediated hypoxic ventilatory response (13). The contribution of orexin to the response was especially strong in the active phase of the circadian cycle, likely because this is the phase in which orexin neurons have high activity (13). More recently, we published data suggesting that orexin augments the peripheral chemoreflex response to hypoxia, potentially through the activation of nTS-projecting CRH neurons (11). We showed that *1*) global orexin receptor (OxR) blockade reduced both the activation (as quantified by c-Fos immunoreactivity) of nTS-projecting CRH neurons and the strength of the reflex and *2*) targeted knockdown of the orexin 1 receptor (Ox1R) in the PVN reduced the activation of the PVN and the strength of the reflex (11). These observations, coupled with previous work from our group, are consistent with the concept that the CRH-nTS pathway contributes significantly to the reflex and that orexin neurons facilitate the reflex through the PVN-nTS pathway. That said, evidence that directly supports these concepts is lacking.

Here we assessed the degree to which nTS-projecting CRH neurons in the PVN contribute to the magnitude of the reflex and whether orexin neurons facilitate the reflex through CRH neurons in the PVN-to-nTS pathway (CRH-nTS). We also evaluated the extent that circadian phase modulates the influence of the CRH-nTS pathway on the reflex. We hypothesized that nTS-projecting CRH neurons are essential for the full peripheral chemoreflex and that orexin facilitates the reflex primarily through the CRH-nTS pathway. Based on our previous findings, we further speculated that the effect of the CRH-nTS pathway and its facilitation by orexin are strongest in the active phase of the circadian cycle.

## MATERIALS AND METHODS

### Ethical Approval

All animal experiments were approved by the University of Missouri Institutional Animal Care and Use Committee (Protocol No. 43650), and experiments were performed in accordance with the American Physiological Society’s Guiding Principles for the Care and Use of Vertebrate Animals in Research and Training and the National Institutes of Health Guide for the Care and Use of Laboratory Animals.

### Animals

All experiments were conducted using adult male Sprague Dawley rats, aged between 2 and 3 mo, and weighing 250–350 g. Rats were purchased from Envigo (Indianapolis, IN) and were housed in a controlled environment with a 12-h light/dark cycle and a room temperature of 22°C. Rats had access to food and water ad libitum. Experiments were conducted during both the inactive and active phases of the diurnal cycle. At the time of experiments and immunohistochemical analyses, the rats were between 3 and 4 mo old.

Rats designated for experiments during the inactive phase were maintained on a standard light cycle, with lights on at 08:00 AM and lights off at 08:00 PM. Experiments were conducted between 12:00 PM and 03:00 PM, corresponding to Zeitgeber time (ZT) 5–8, which translates to 4–7 h into the inactive phase. For experiments during the active phase, rats were acclimated to a room where the light cycle was shifted by 12 h, with lights turned on at 08:00 PM and turned off at 08:00 AM. Rats were given 1 wk to acclimate to their home room with the lights reversed. Experiments were also conducted between 12:00 PM and 3:00 PM, corresponding to ZT 16–19, ∼4–7 h into the active phase. During active-phase experiments, laboratory lights were turned off, and workstations were illuminated with red light to minimize disruption to the rats’ natural circadian rhythms.

### Viral Targeting of the CRH-nTS Pathway

#### PVN nanoinjection.

**Figure 1.**
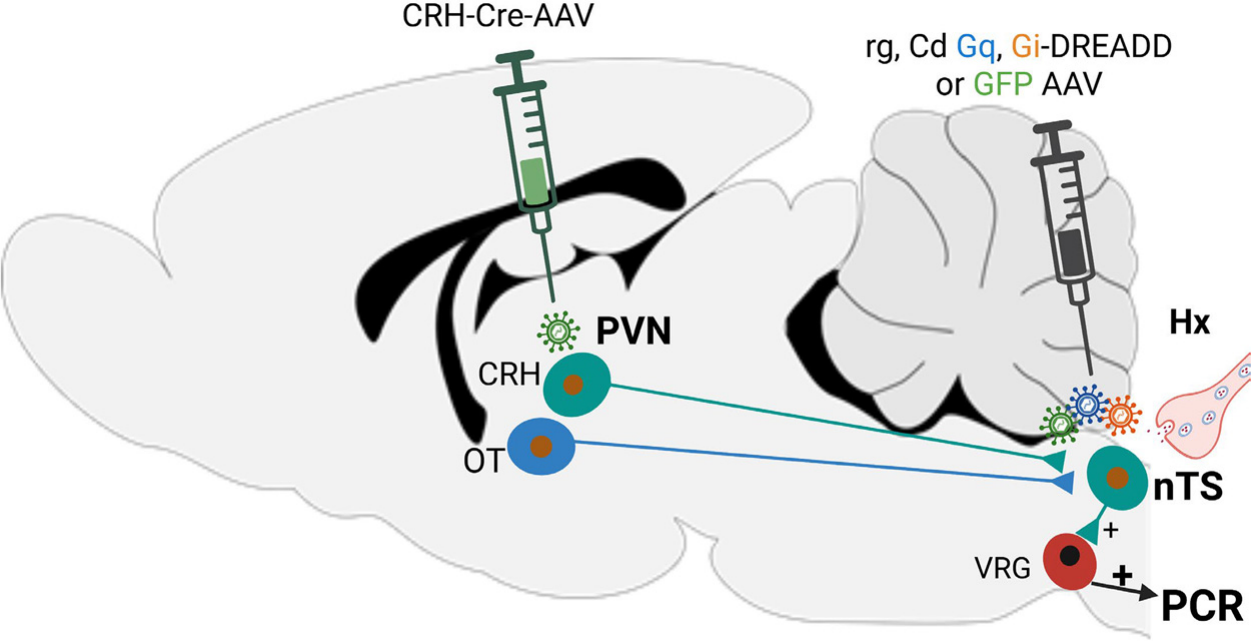
Schematic of strategy targeting CRH-nTS pathway. A Cre-expressing AAV driven by the CRH promoter (CRH-Cre-AAV) was injected into the PVN. Following 1 wk recovery, one of three Cre-dependent (Cd), retrogradely-transported (rg) AAVs was injected into the nTS: *1*) AAV expressing Gq-Designer Receptor Exclusively Activated by Designer Drug (Gq-DREADD); *2*) AAV expressing Gi-DREADD, or *3*) AAV expressing GFP alone with no DREADD. Strategy allows for chemogenetic activation (Gq) or inhibition (Gi) of CRH neurons projecting to nTS to interrogate their influence on strength of the peripheral chemoreflex (PCR) in response to acute hypoxia (Hx). CRH, corticotropin-releasing hormone; GFP, green fluorescent protein; nTS, nucleus of the solitary tract; PVN, paraventricular nucleus; VRG, ventral respiratory group.

Our overall strategy to target nTS-projecting CRH neurons is shown in Fig. 1. An adeno-associated viral (AAV) vector driving the expression of the optimized Cre recombinase (Cre) gene behind the full-length rat CRH promoter (AAV2-CRH-Cre, Genedetect, 1 × 10^13^ vg/mL) was used to target Cre expression specifically to CRH neurons in the PVN. Rats were deeply anesthetized with isoflurane (5% for induction; 2%–2.5% for maintenance, Aerane; Baxter, Deerfield, IL) and secured in a stereotaxic apparatus (Kopf Instruments, Tujunga, CA). Anesthesia was monitored throughout the surgery and was assessed approximately every 15 min by responses to toe pinch and absence of the palpebral reflex. A midline incision was made along the dorsal surface of the skull, and the muscle and fascia were dissected bluntly to expose bregma and lambda. The head position was adjusted to ensure that bregma and lambda were aligned in the same horizontal plane. A small hole was drilled in the skull to expose the surface of the brain. Glass micropipettes containing AAV-CRH-Cre were nanoinjected bilaterally into the PVN (*n* = 20; 200 nL each side), over a period of 1 min, using the following coordinates: 1.8–2.0 mm caudal to bregma, 0.5 mm lateral from the midline, and 7.6–7.8 mm ventral to the dura. The pipette remained in the tissue for 5 min to minimize movement of AAV up the injection tract. The pipette was then removed, and the incision site was closed. Rats were treated postoperatively with fluids (3 mL 0.9% saline sc), enrofloxacin (5 mg/kg sc), and carprofen (5 mg/kg sc), for hydration, prevention of infection, and pain management, respectively, as supportive therapy after surgery. Following the termination of anesthesia, rats were monitored until upright and alert and then returned to their cages. Animals were allowed 1–2 wk for recovery and for expression of Cre recombinase AAV in the CRH neurons.

#### nTS nanoinjection.

Rats with prior PVN nanoinjection of AAV-CRH Cre (∼10 days after injection) were deeply anaesthetized, placed in a stereotaxic apparatus (Kopf Instruments), and monitored, as mentioned earlier. The dorsal brainstem was exposed via a limited occipital craniotomy. Glass micropipettes contained one of the following retrogradely transported AAVs that expressed a DREADD sequence and mCherry [or control expressing solely enhanced green fluorescent protein (EGFP)] behind the human synapsin promoter (hSyn) to target neurons: *1*) AAV2 coupled to an inhibitory DREADD (Cre-Gi-DREADD:AAV2rg-hSyn-DIO-hM4DmCherry; Addgene #44362-AAV2; 5 × 10^12^ vg/mL) (*n* = 8); *2*) AAV2 coupled to an excitatory DREADD (Cre-Gq-DREADD:AAV2rg-hSyn-DIO-hM3D-mCherry; Addgene #44361-AAV2, 6 × 10^12^ vg/mL) (*n* = 8), or *3*) control AAV2 lacking a DREADD sequence that expresses only EGFP (Cre-EGFP: AAV2rg-hSyn-DIO:EGFP; Addgene #50459-AAV2, 4 × 10^12^ vg/mL) (*n* = 4). Micropipettes were advanced into the nTS, and 90 nL of AAV was injected bilaterally using the following coordinates for the nTS: 0.4 mm rostral and ± 0.4 lateral from the calamus scriptorius (CS), and 0.4 mm ventral from the surface. This location receives heavy innervation from carotid body afferents and receives projections from CRH neurons. The pipette remained in the brain for 5 min. Following the surgery, the incision site was closed, and rats received postoperative care as mentioned earlier. Once awake and alert, rats were returned to their home cages and allowed a period of 2 wk for recovery after surgery and for adequate DREADD expression before the day of the experiments. We removed one rat from Gi-DREADD group and two rats from the Gq-DREADD group due to poor health following nTS injection.

### Whole Body Plethysmography

We used whole body plethysmography to monitor respiratory and metabolic variables during experiments, assessing the acute hypoxic ventilatory response and for exposing rats to 2 h of hypoxia or normoxia for future immunohistochemical analyses. The volume of the chamber used was ∼15 L. Gas, either room air or hypoxia, was delivered to the chamber via pressurized cylinders. The pressure within the chamber was maintained near atmospheric levels using a standard laboratory bench vacuum. Chamber temperature and humidity were continually monitored, with values recorded for each rat to calculate tidal volume via the approach described by Drorbaugh and Fenn (14). This calculation also requires calibration injections of a known volume (1 mL), which was performed for each rat at the end of the experiment. All cardiorespiratory variables were monitored in real time using LabChart v8 (ADInstruments).

### Chemoreflex Testing in Conscious Animals

We tested the peripheral chemoreflex in rats 2 wk following the second AAV microinjections to the nTS. All experiments were performed during both the active and inactive phases in each rat. The plethysmography chamber was maintained at ∼25°C throughout the experiment. Normoxic gas (21% O_2_, balance N_2_) or anoxic gas (97% N_2_, 3% CO_2_) was delivered to the chamber from premixed cylinders, as previously described (13). The small amount of CO_2_ added to the hypoxic gas was used to minimize the hyperventilation-induced washout of CO_2_ and fall in arterial Pco_2_, as we have previously verified (13). After a 15-min settling period breathing normoxic gas, a baseline recording was made over 30 min with the gas supply coming from the normoxic cylinder. To test the peripheral chemoreflex, the supply of gas to the chamber was switched from the normoxic to the anoxic cylinder using a Hypoxydial (STARR Life Sciences, Oakmont, PA), inducing a rapid, ramp-like fall in inspired O_2_ from 21% to 10% and an increase in inspired CO_2_ from 0 to appoximately 2.5%–3%. The entire ramp protocol occurred over a period of appoximately 5–10 min. The rapid washin time of the hypoxic gas effectively isolates the peripheral chemoreflex while avoiding secondary effects of hypoxia on metabolic rate or body temperature (15). To assess the effect of either activation or inhibition of nTS-projecting CRH neurons on the reflex responses, rats were first injected with vehicle [dimethyl sulfoxide (DMSO)-saline mix; intraperitoneally], followed by reflex testing 30 min later. After a 30-min recovery period, rats were injected with Compound 21 [C21; Tocris, Minneapolis, MN; 1 mg/kg in DMSO-saline mix; intraperitoneally—dose based on our previous study (6)], the selective agonist for DREADDs followed by a second reflex test 30 min later. After another 30 min recovery period, rats were injected with SB-334867, a selective Ox1R antagonist [Tocris; 1 mg/kg ip—dose based on the original characterization of the antagonist (16)], followed by a third reflex test. We have previously shown that multiple exposures to brief hypoxia have no significant effect on the strength of the reflex (6, 7).

#### Immunohistochemistry to assess targeting of the CRH-nTS pathway and effects of DREADD-based manipulations.

We aimed to quantify the degree to which we successfully targeted nTS-projecting CRH neurons with Gq- (*n* = 9) or Gi-DREADDs (*n* = 8) (i.e., by quantifying the coexpression of neuronal mCherry or GFP, and CRH) and to assess the degree that DREADD activation influenced the hypoxia-induced activation of nTS-projecting CRH neurons (via c-Fos immunoreactivity).

We first acclimated rats to the whole body plethysmography chamber for 2 h per day over 2 consecutive days. The acclimation process also included intraperitoneal injections of normal saline on each day. The following day, rats were placed in the chamber and given 30 min for acclimation in room air. Subsequently, the rats received an intraperitoneal injection (1 mL) of either saline or C21 (1 mg/kg), and a 30-min period was allowed for DREADD activation. The gas mixture was then adjusted, and groups of rats were exposed to hypoxia (Hx; holding inspired O_2_ at 10%–11% O_2_, with 2%–3% CO_2_) for 2 h during the light (inactive) phase (ZT 5–8). Animal numbers: CRH-Cre-Gq-DREADD: vehicle (*n* = 5); C21 (*n* = 4); CRH-Cre-Gi-DREADD: vehicle (*n* = 4); C21 (*n* = 4). Rats were exposed to hypoxia for 2 h to ensure adequate time for robust expression of c-Fos (5, 17). Our group and others have published multiple studies showing that hypoxia leads to activation of the PVN, when compared with animals breathing normoxia (5, 10, 18). Thus, for simplicity, we did not include normoxic controls in these evaluations.

Immediately after hypoxic exposure, rats were deeply anesthetized with 5% isoflurane followed by transcardial perfusion. Cold-heparinized phosphate buffer solution (PBS; ∼500 mL, pH 7.4, 10 IU/mL heparin) was first perfused, followed by 500 mL of cold 4% paraformaldehyde (PFA, pH 7.2). After the perfusion, the brains were harvested, postfixed overnight in 4% PFA, and stored in PBS. Coronal sections of the forebrain (40 μm) containing the PVN were cut using a vibratome (1000S, Leica, Germany) with tissues stored in cryoprotectant until immunohistochemistry (IHC) was performed. One in every six consecutive sections was processed for IHC. All procedures were carried out using protocols similar to those previously described (5, 19), and all antibodies were verified in previously published studies (5, 10, 13, 19–22). Sections were rinsed with 0.01 M phosphate-buffered saline (3 × 10 min PBS), blocked in 10% normal donkey serum (Millipore, S30) in 0.3% Triton-0.01 M PBS (PBS-T), and incubated overnight in 3% normal donkey serum and 0.3% PBS-T containing the primary antibodies. c-Fos was detected using rabbit anti-c-Fos (1:3,000, ab190289, Abcam). Sections from the hypothalamus were incubated with guinea pig anti-CRH (1:500, T-5007, Peninsula Laboratories). All sections were also stained for mCherry using chicken anti-mCherry; (1:500, ab205402, Abcam, Waltham, MA; RRID: AB_2722769). After 24 h with primary antibodies, sections were rinsed in PBS and incubated for 2 h with appropriate secondary antibodies conjugated to Cy2, Cy3, or Cy5 (1:200, Jackson ImmunoResearch), with 1% normal donkey serum in 0.3% Triton-0.01 M PBS. Sections were then rinsed and mounted on gel-coated slides and cover slipped with ProLong Diamond (Thermo Fisher Scientific, P36970).

### Microscopy and Image Analysis

A rat brain atlas was used to determine the appropriate levels, relative to bregma, within the hypothalamus and brainstem (23). For all animals, sections containing the hypothalamus were examined using a fluorescence microscope (BX51; Olympus) equipped with a digital monochrome camera (ORCA-ER; Hamamatsu) and a spinning disk confocal unit (Olympus). Each image consisted of stacks of 11 optical planes (2 μm between planes). Image stacks were imported into ImageJ (v.1.48v). Quantification of positively labeled cells from each of the immunohistochemical protocols was performed using ImageJ and adjusted for contrast and brightness only. We examined sections from animals exposed to hypoxia after either intraperitonial injection of C21 or vehicle.

#### PVN analysis.

We quantitatively assessed neuronal activation (immunoreactivity for c-Fos) in the PVN of rats that were exposed to hypoxia after systemic injection of either vehicle or C21, at three different levels: −1.7, −1.9, and −2.1 mm from bregma. Unilateral counts were averaged at each level across all animals within each group. CRH neurons exhibited bright cytosolic labeling with a blank nuclear region. In Gi-DREADD and Gq-DREADD rats, unilateral counts of retrogradely mCherry-labeled CRH neurons (i.e., those CRH neurons projecting to nTS) with c-Fos-IR were performed at the same levels relative to bregma. Cells were considered double-labeled when they met the criteria for both retrograde mCherry labeling and c-Fos-IR under more than one filter set in the same focal plane. Cells that showed a positive signal under all three filter sets (retrograde mCherry, c-Fos-IR, and CRH labeling) were considered triple-labeled.

### Data and Statistical Analyses

To assess the degree to which the DREADD-mediated activation or inhibition of nTS-projecting CRH neurons influenced the hypoxic ventilatory response, we calculated ventilatory frequency (*f*), tidal volume (V_T_), minute ventilation (V̇e), and the ventilatory equivalent [V̇e/metabolic CO_2_ production (V̇co_2_)] during 1 min of the normoxic period immediately before hypoxia exposure. We also measured these variables across 10–20 breaths, uncontaminated by movement artifact, when the inspired O_2_ was 16%, 14%, 12%, 10%, and 8% (±0.5% at each level), as we have done in previous studies (13). Initial findings indicated that pathway silencing reduced hypoxic responses across all levels of hypoxia (Supplemental Fig. S1). For simplicity and based on these and our previously published data (13), for each rat we subsequently analyzed the hypoxic responses at only one level of hypoxia (10% inspired O_2_).

All statistical analyses were conducted using GraphPad Prism (v.8.0.2; GraphPad Software, San Diego, CA). Two-way ANOVA with repeated measures was used to examine the effects of O_2_ level (normoxia and hypoxia) and drug (Veh, C21, and SB-334867) on respiratory variables. Two-tailed Student’s *t* tests were used to evaluate the effects of either C21 or vehicle on the percentage of mCherry-labeled CRH neurons displaying c-Fos-IR. Post hoc analysis [Fisher’s least significant difference (LSD) test] was conducted to examine specific differences among groups when appropriate. Differences were considered significant at *P* ≤ 0.05. All values are expressed as means ± SD. Details of the statistical analyses are provided in the figure legends.

## RESULTS

### No Influence of C21 on the Peripheral Chemoreflex in the Absence of DREADD Expression

**Figure 2.**
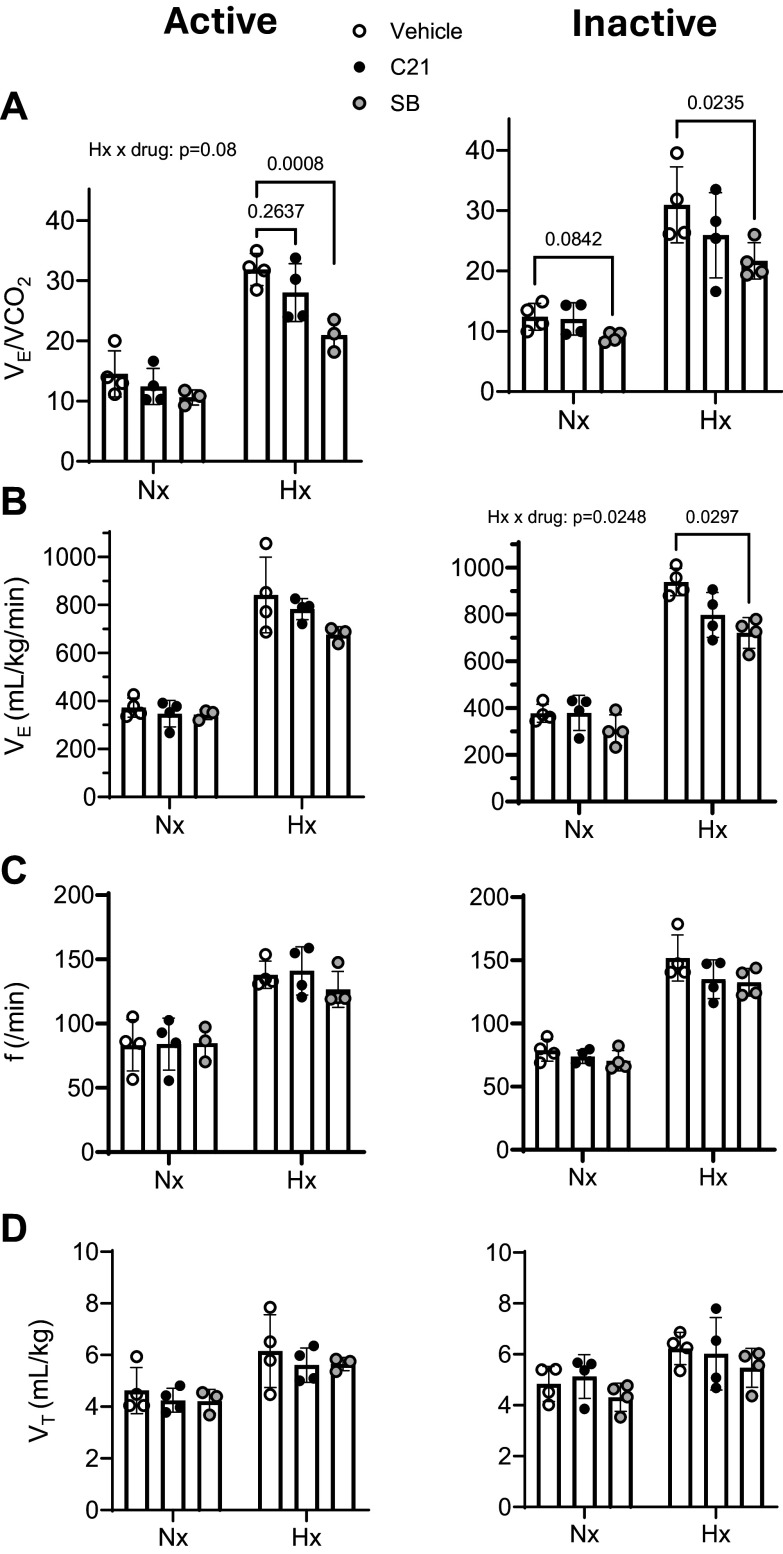
No influence of C21 on the peripheral chemoreflex in the absence of DREADD expression. Shown are ventilatory equivalent (V̇e/V̇co_2_) (*A*), ventilation (V̇e) (*B*), respiratory frequency (*f*) (*C*), and tidal volume (V_T_) (*D*) in the active (*left*) and inactive phases (*right*), under baseline, normoxic (Nx), and hypoxic (Hx) conditions (*n* = 3 or 4). Administration of C21 in the absence of DREADD expression had no significant effects on respiratory variables and did not prevent an effect of Ox1R blockade with SB-334867 (SB) on the hypoxic ventilatory response. Values are presented as averages ± SD. *P* values for interactions and post hoc analyses are indicated. C21, Compound 21.

We first examined whether the administration of C21, in the absence of DREADD expression, had any influence on the ventilatory response to 10% inspired O_2_. To this end, we tested four rats that had CRH-Cre AAV injected into the PVN and a retrogradely transported AAV expressing only EGFP (and no DREADD) into the nTS. In both the active and inactive phases, the application of C21 did not influence any measured respiratory variables, whereas Ox1R blockade reduced V̇e/V̇co_2_ responses to hypoxia (Fig. 2*A*). Thus, the reflex was left unperturbed by C21 administration to rats that had AAVs injected that did not express DREADDs. In addition, in the absence of CRH-nTS pathway silencing, Ox1R signaling makes a significant contribution to the reflex, as we have previously demonstrated.

### CRH-nTS Pathway Inhibition Reduces the Strength of the Peripheral Reflex Exclusively in the Active Phase and Occludes the Effect of Ox1R Blockade on Reflex Strength

We then proceeded to test the degree that *1*) the CRH-nTS pathway influences the peripheral chemoreflex; *2*) Ox1R-mediated drive through the CRH-nTS pathway bolsters the reflex, and *3*) whether the influence of the pathway and its modulation by orexin depend on circadian phase.

**Figure 3.**
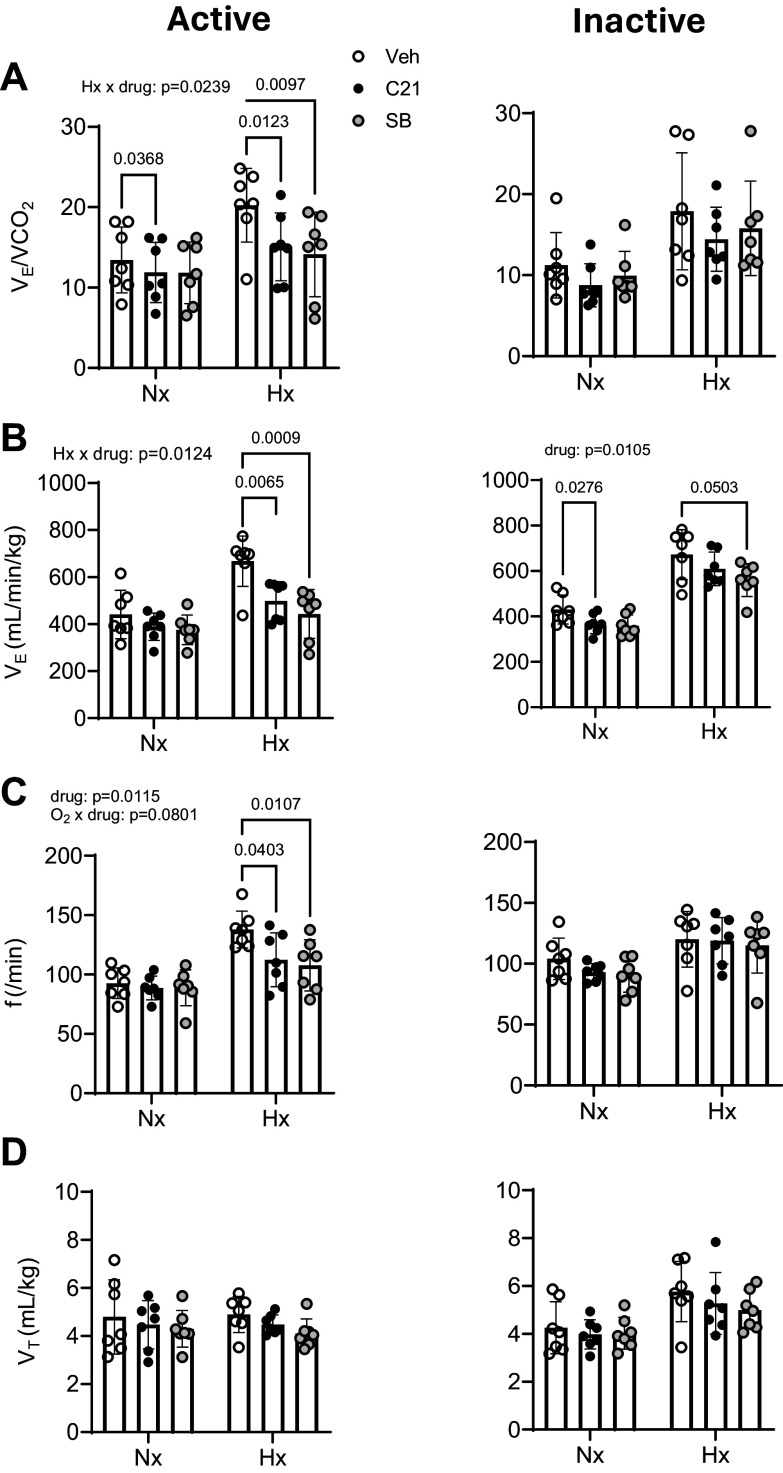
Gi-DREADD inhibition of the CRH-nTS pathway reduced the strength of the peripheral chemoreflex. Shown are ventilatory equivalent (V̇e/V̇co_2_) (*A*), ventilation (V̇e) (*B*), respiratory frequency (*f*) (*C*), and tidal volume (V_T_) (*D*) under baseline, normoxic (Nx), and hypoxic (Hx) conditions in the active (*left*) and inactive phases (*right*) (*n* = 7). Silencing the CRH-nTS pathway (C21) in the active phase significantly reduced V̇e/V̇co_2_ in Nx and especially in Hx (Hx × drug: *P* = 0.0239). In Hx, the suppressive effects of silencing were due to a reduction in V̇e (Hx × drug: *P* = 0.0124) because of a reduction in f (*P* = 0.0495). In the active phase, pathway silencing occluded the effect of Ox1R blockade with SB-334867 (SB). Values are presented as averages ± SD. *P* values for interactions and post hoc analyses are indicated. C21, Compound 21; CRH, corticotropin-releasing hormone; nTS, nucleus of the solitary tract.

**Figure 4.**
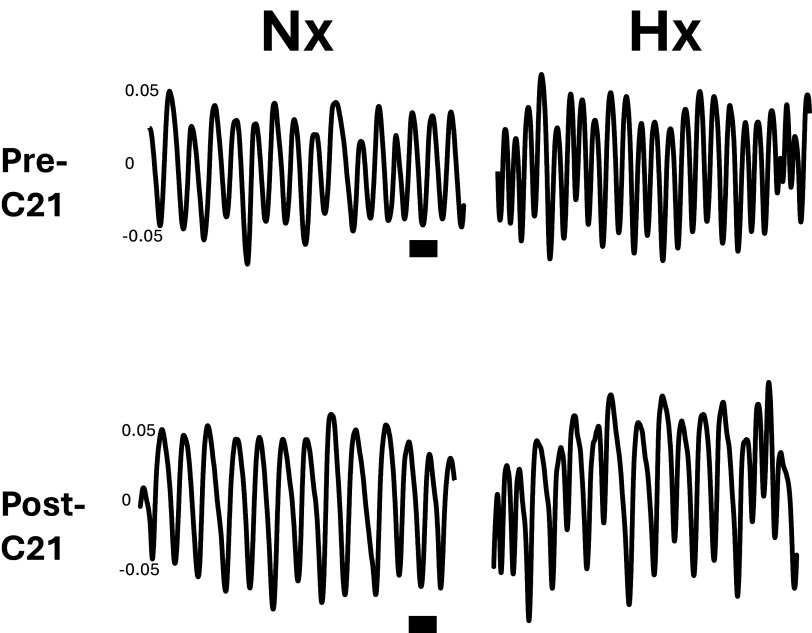
CRH-nTS pathway silencing reduces the respiratory frequency response to hypoxia. Shown are raw tracings of breathing in normoxia (Nx) and hypoxia (Hx), before and after administration of C21. Note the reduced frequency response to Hx. Raw voltage over time is shown. Scale bar = 1 s. C21, Compound 21.

In the active phase, silencing the CRH-nTS pathway significantly reduced V̇e/V̇co_2_ during baseline room air conditions, and at more than one level of hypoxia, as shown in Supplemental Fig. S1. There was a larger suppressive effect of silencing during hypoxia when compared with room air (Supplemental Fig. S1 and Fig. 3*A*, *left*). The blunted V̇e/V̇co_2_ response following pathway silencing was due to a reduced ventilatory response to hypoxia (Supplemental Fig. S1 and Fig. 3*B*, *left*), whereas V̇co_2_ was uninfluenced by pathway silencing (V̇co_2_ before silencing: 34 ± 4 mL O_2_/min/kg; after silencing: 34 ± 7 mL O_2_/min/kg). The reduced ventilatory response to hypoxia following pathway silencing in the active phase was mediated by a significantly reduced frequency response to hypoxia (Fig. 3*C*, *left*), with no effect of silencing on V_T_ (Fig. 3*D*, *left*). Raw respiratory traces demonstrating the effect of pathway silencing on the respiratory frequency response to hypoxia in the active phase are shown in Fig. 4.

In contrast to pathway silencing in the active phase, silencing in the inactive phase had no effect on the increased V̇e/V̇co_2_ in response to hypoxia [compare *left* panel of Fig. 3*A* (active phase) with the *right* panel (inactive phase)]. However, there was a small yet statistically significant effect of pathway silencing on V̇e in normoxia (Fig. 3*B*, *right*). As this was not reflected in a decrease in the ventilatory equivalent during normoxia, the effect of silencing on V̇e was likely secondary to a small decrease in V̇co_2_ during normoxia.

To provide insight into the extent that orexin signals via the CRH-nTS pathway to augment the reflex, we performed a third reflex test following the blockade of Ox1R during pathway inhibition. Following pathway silencing, systemic application of SB-334867 had no further inhibitory effects on any respiratory variables in either normoxia or hypoxia. For example, compared with its level after silencing, Ox1R blockade in the active phase left V̇e/V̇co_2_ unchanged (Fig. 3*A*, *left*). This was also the case for the V̇e (Fig. 3*B*, *left*) and *f* (Fig. 3*C*, *left*) responses to hypoxia. Taken together, these data suggest that the full strength of the peripheral reflex relies on the CRH-nTS pathway and that orexin neurons predominantly rely on the CRH-nTS pathway for facilitating the reflex.

### DREADD-Based Activation of the CRH-nTS Pathway Has No Influence on the Strength of the Peripheral Chemoreflex

**Figure 5.**
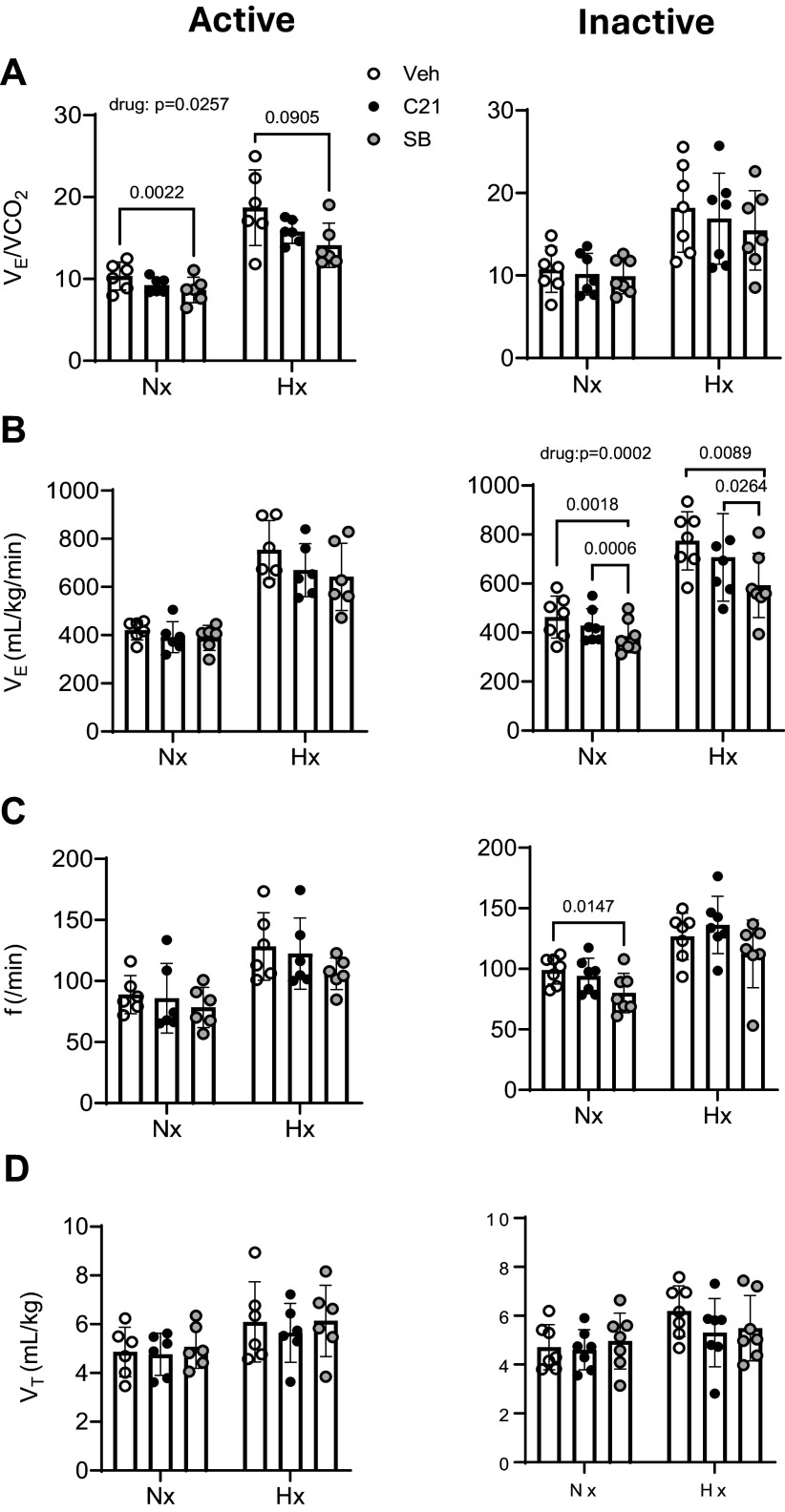
Gq-DREADD excitation of the CRH-nTS pathway had no influence of the strength of the peripheral chemoreflex. Shown are ventilatory equivalent (V̇e/V̇co_2_) (*A*), ventilation (V̇e) (*B*), respiratory frequency (*f*) (*C*), and tidal volume (V_T_) (*D*) under baseline, normoxic (Nx), and hypoxic (Hx) conditions in the active (*left*) and inactive phases (*right*) (*n* = 6). Excitation of the CRH-nTS pathway (C21) had no significant influence on V̇e/V̇co_2_ in Nx or Hx but, in the active phase, did not prevent an effect of Ox1R blockade with SB-334867 (SB) (drug: *P* = 0.0257). Values are presented as averages ± SD. *P* values for interactions and post hoc analyses are indicated. C21, Compound 21; CRH, corticotropin-releasing hormone; nTS, nucleus of the solitary tract.

In addition to silencing the CRH-nTS pathway with Gi-DREADDs, we also used Gq-DREADDs to allow activation of the pathway before and during hypoxia. Unlike silencing, activation of the CRH-nTS pathway in either the active or inactive phase had no influence on baseline V̇e/V̇co_2_ or its response to hypoxia. In addition, the activation of the pathway did not occlude an inhibitory influence of Ox1R blockade on normoxic V̇e/V̇co_2_ or its tendency to reduce the V̇e/V̇co_2_ response to hypoxia in the active phase (Fig. 5*A*, *left*). In the inactive phase, there was no influence of Ox1R blockade on V̇e/V̇co_2_ in normoxia or hypoxia. However, following pathway activation, Ox1R blockade significantly reduced V̇e in normoxia and hypoxia (Fig. 5*B*, *right*), due to a decrease in *f* (Fig. 5*C*, *right*). These effects were likely due to an effect of Ox1R blockade on V̇co_2_, given that there was no effect of blockade on V̇e/V̇co_2_.

### Confirmation of Selective Targeting of CRH-nTS Pathway and Consequences of DREADD Activation on Its Hypoxia-Induced Excitation

**Figure 6.**
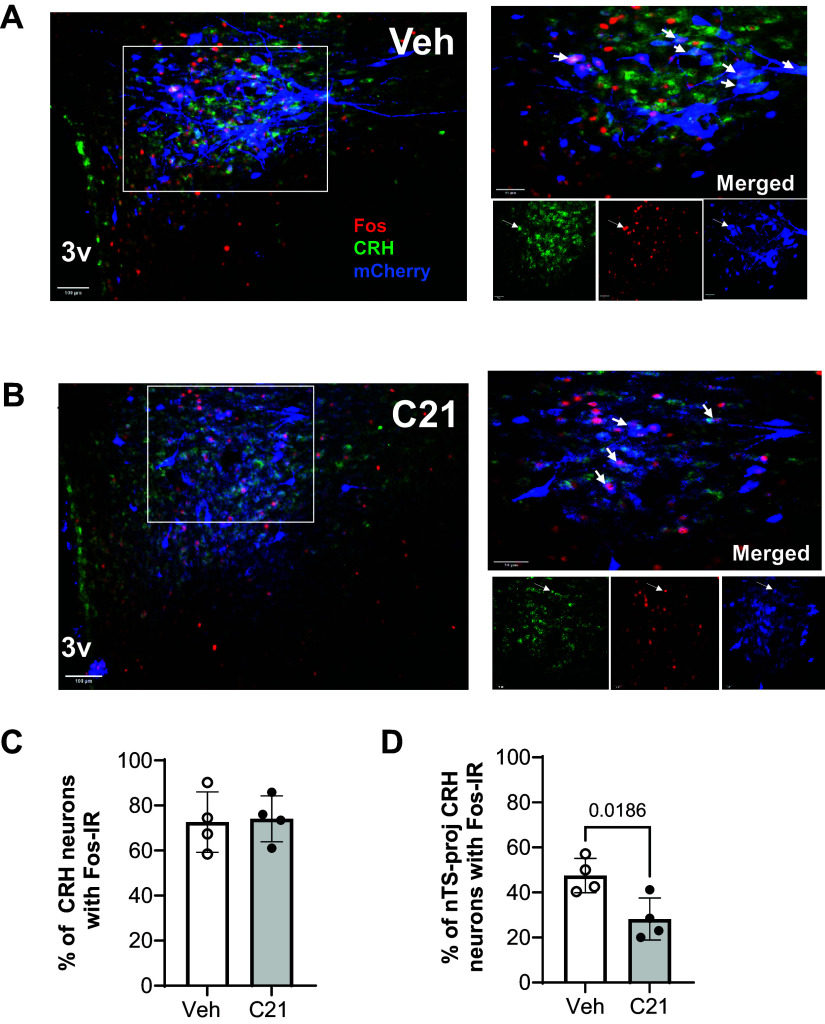
Confirmation that Gi-DREADD-mediated pathway inhibition reduced the number of hypoxia-activated (c-Fos immunoreactive), nTS-projecting CRH neurons. Images show immunohistochemical staining for c-Fos (red nuclei), CRH neurons (green), and mCherry (blue) in the PVN of rats with Gi-DREADDs following administration of either vehicle (*A*) or C21 (*B*) prior to exposure to hypoxia. mCherry confirms DREADD expression in PVN CRH neurons. Magnification of boxed region and individual channels shown in panels on the *right*. Arrows in merged and individual channels indicate neurons immunoreactive for all 3 targets. *C*: % of CRH neurons immunoreactive for c-Fos. *D*: % of nTS-projecting CRH neurons immunoreactive for c-Fos. Values are presented as averages ± SD. *P* value indicates significant difference between vehicle and C21 on the number of projecting, activated CRH neurons. C21, Compound 21; CRH, corticotropin-releasing hormone; nTS, nucleus of the solitary tract; PVN, paraventricular nucleus.

**Figure 7.**
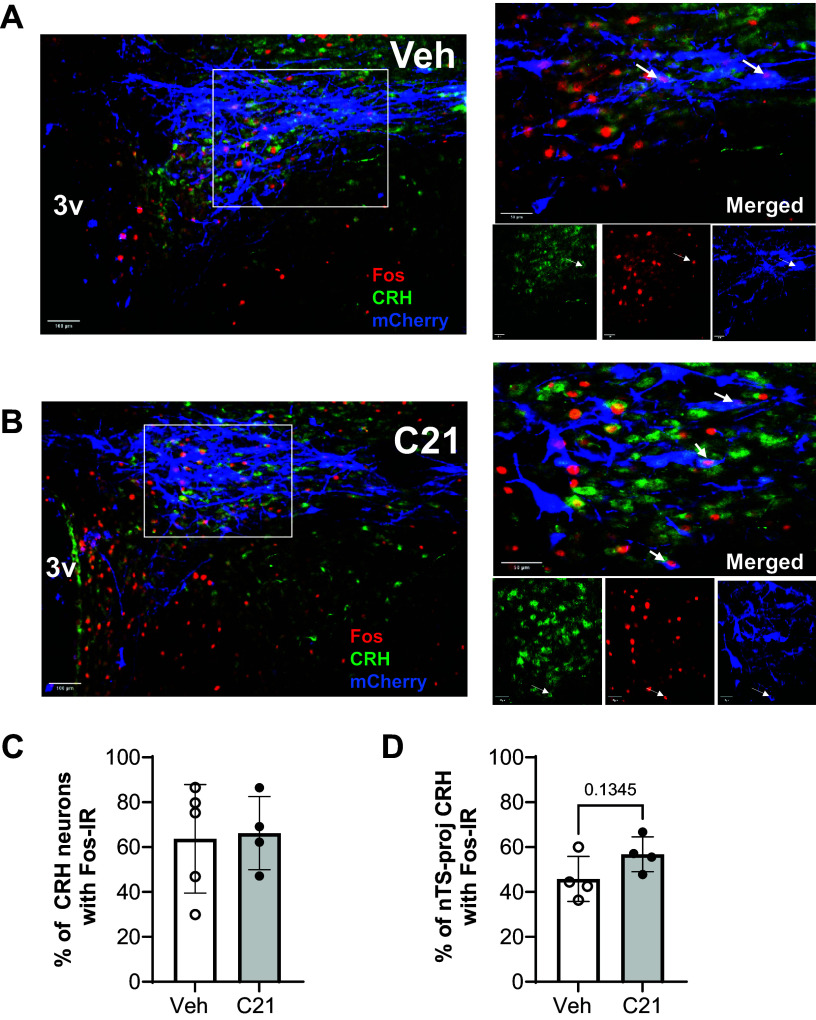
Confirmation that Gq-DREADD-mediated pathway activation did not alter the number of hypoxia-activated, nTS-projecting CRH neurons. Images show immunohistochemical staining for c-Fos (red nuclei), CRH neurons (green), and mCherry (blue) in the PVN of rats with PVN Gq-DREADDs following administration of either vehicle (*A*) or C21 (*B*) before exposure to hypoxia. mCherry confirms DREADD expression in PVN CRH neurons. Magnification of boxed region and individual channels shown in panels on the *right*. Arrows in merged and individual channels indicate neurons immunoreactive for all 3 targets. *C*: % of CRH neurons immunoreactive for c-Fos. *D*: % of nTS-projecting CRH neurons immunoreactive for c-Fos. Values are presented as averages ± SD. ns, no significant difference between vehicle and C21 on the number of projecting, activated CRH neurons. C21, Compound 21; CRH, corticotropin-releasing hormone; nTS, nucleus of the solitary tract; PVN, paraventricular nucleus.

Our approach involved the injection of a CRH-driven Cre AAV into the PVN coupled with the injection of retrogradely transported, Cre-dependent mCherry and DREADD-expressing AAV in the nTS. Thus, only CRH neurons projecting to the nTS should express the DREADDs and mCherry. Using IHC, we were able to identify mCherry expression in the PVN of all nine rats that had Gq-expressing AAVs injected into nTS and all eight rats that had Gi-expressing AAVs injected in nTS. Example images are shown in Fig. 6 (Gi) and Fig. 7 (Gq) from (at −1.9 mm from bregma). For both Gq and Gi groups, approximately 93%–96% of mCherry-expressing neurons (i.e., those neurons expressing Gq or Gi DREADDs) also had detectable CRH (Tables 1 and 2).

**Table 1.** Number of CRH neurons activated by hypoxia (+Fos) and/or that project (+mCh) to nTS following vehicle or C21-induced inhibition of the CRH-nTS pathway

Group	No. of CRH	No. of CRH + Fos	No. of CRH + Fos/No. of CRH (%)	No. of mCh	No. of CRH + mCh	No. of CRH + mCh/No. of mCh (%)	No. of CRH + mCh + Fos	No. of CRH + mCh + Fos/No. of CRH + mCh (%)
Veh	108 ± 16	74 ± 18	69 ± 12	22 ± 24	20 ± 22	94 ± 6	9 ± 9	50 ± 8
C21	122 ± 18	87 ± 18	72 ± 10	17 ± 3	15 ± 2	93 ± 6	5 ± 2	29 ± 11*

C21, Compound 21; CRH, corticotropin-releasing hormone; nTS, nucleus of the solitary tract. *Significantly different from Veh.

**Table 2.** Number of CRH neurons activated by hypoxia (+Fos) and/or that project (+mCh) to nTS following vehicle or C21-induced activation of the CRH-nTS pathway

Group	No. of CRH	No. of CRH + Fos	No. of CRH + Fos/No. of CRH (%)	No. of mCh	No. of CRH + mCh	No. of CRH + mCh/No. of mCh (%)	No. of CRH + mCh + Fos	No. of CRH + mCh + Fos/No. of CRH + mCh (%)
Veh	149 ± 76	100 ± 69	66 ± 24	24 ± 16	22 ± 16	93 ± 8	8 ± 3	44 ± 8
C21	96 ± 6	60 ± 8	66 ± 1	28 ± 6	27 ± 4	96 ± 6	15 ± 1	57 ± 7

C21, Compound 21; CRH, corticotropin-releasing hormone; nTS, nucleus of the solitary tract, *significantly different from Veh.

We also examined the proportion of CRH neurons that expressed c-Fos following hypoxia (indicating activation) and the degree to which Gq- or Gi-DREADD activation altered the number of nTS-projecting CRH neurons that were also immunoreactive for c-Fos following hypoxia. Before hypoxia, C21 or vehicle alone was administered to rats expressing Gi-DREADDs in nTS-projecting CRH neurons. Compared with control, Gi-DREADD-mediated inhibition of the CRH-nTS pathway had no significant influence on the activation of CRH neurons globally; in both groups, ∼70% of the total population CRH neurons also expressed c-Fos following hypoxia (Table 1, Fig. 6*C*). However, compared with control, pathway silencing reduced the number of activated nTS-projecting CRH neurons, a small fraction of the total number of CRH neurons, by 50% (Table 1, Fig. 6*D*).

Gq-DREADDs were abundantly expressed in CRH neurons (Fig. 7, *A* and *B*). However, the excitation of CRH neurons via Gq-DREADDs had no significant influence on the proportion of CRH neurons activated by hypoxia globally (Fig. 7*C*). Although there was a trend for an increased proportion of nTS-projecting CRH neurons with c-Fos expression following Gq-mediated activation, this effect did not reach statistical significance (*P* = 0.1345; Fig. 7*D*).

## DISCUSSION

Ours is the first study to show that nTS-projecting CRH neurons are necessary for the full expression of the peripheral chemoreflex. We have also provided evidence that orexin, acting at Ox1R, facilitates the reflex via the CRH-nTS projection. Previous work has demonstrated that, globally, PVN neurons are required for the full reflex (7, 8). Inhibiting the PVN also compromises the full activation (c-Fos immunoreactivity) of the nTS (7), suggesting, albeit indirectly, that a PVN-to-nTS circuit is activated by hypoxia to facilitate the reflex. More conclusive evidence for this concept came from experiments showing that specific silencing of only those PVN neurons projecting to the nTS reduced the reflex (6). This effect was of a similar magnitude to global PVN silencing, suggesting that most, if not all, of the influence of the PVN on the hypoxic ventilatory response is mediated via projections to the nTS. Of note, these studies—global inhibition of the PVN and specific inhibition of the PVN-nTS projection—were conducted only in the inactive phase and did not assess the contribution of specific PVN neuronal phenotypes.

### CRH Neurons Projecting to nTS Facilitate the Peripheral Chemoreflex

In the current study, the silencing of the CRH-nTS projection reduced *f*, V̇e_,_ and V̇e/V̇co_2_ in the active phase during normoxic and hypoxic conditions, with a more pronounced effect in hypoxia. This work extends our previous findings showing that hypoxia activates (i.e., c-Fos) the CRH-nTS projection (5, 11). The current data suggest that of all the nTS-projecting PVN neurons that project to the nTS, those immunoreactive for CRH are the dominant contributors to the hypoxic ventilatory response. As with silencing the entire nTS projection (6), specific silencing of the CRH-nTS pathway reduced predominantly the respiratory frequency response to hypoxia, but not the tidal volume response. However, under control (vehicle) conditions, nearly all of the response to hypoxia was mediated by an increase in respiratory frequency, a common observation (24). The effect of CRH-nTS silencing on the ventilatory response to hypoxia was associated with a 50% reduction in the number of nTS-projecting CRH neurons activated by hypoxia. Despite fewer activated, projecting CRH neurons, the total number (projecting and nonprojecting) of CRH neurons activated by hypoxia was not influenced by pathway silencing. Thus, CRH neurons not projecting to the nTS—perhaps those neuroendocrine CRH neurons projecting to the median eminence to elicit HPA activation, or others that project to the spinal cord—were likely activated concurrently with inhibition of the nTS-projecting CRH neurons.

There was no influence of CRH-nTS blockade on the strength of the reflex in the inactive phase. Nevertheless, our previous studies showed that silencing the entire PVN projection to the PVN blunts the reflex in the inactive phase (6). In these earlier studies, we used 5-min exposure to hypoxia rather than the ramp protocol and did not provide supplemental CO_2_ during hypoxia exposure, and it is possible that these differences affected the overall role of this pathway. Alternatively, the current findings could suggest that a distinct PVN-nTS pathway contributes to the strength of the reflex in the inactive phase. Oxytocin (OT) neurons are possible candidates, as they project to the nTS and are strongly activated by hypoxia (5, 11). nTS-projecting OT neurons may contribute to the reflex in either phase.

Gq-mediated activation of CRH-nTS pathway did not influence the strength of the peripheral chemoreflex. It may be that during hypoxia the CRH-nTS pathway is maximally activated, preventing additional chemogenetic excitation. Another possibility is that at this level of hypoxia (10%), second-order nTS neurons are already fully activated by afferents originating in the carotid body chemoreceptors and excitatory neuromodulation from other sources. These data are consistent with our previous studies, showing only a small effect of chemogenetic activation of the entire PVN-nTS projection on reflex strength, which occurred at milder levels of hypoxia (6). Thus, a lack of an effect here*—*i.e., on just the CRH-nTS component of the projection—is perhaps not surprising.

Our findings here are consistent with our group’s previous research using immunohistochemistry and neural tract tracing, which implied an important role for CRH neurons on the hypoxic ventilatory response. We showed that most CRH neurons in the PVN, especially in more caudal aspects of the parvocellular subnuclei, are activated by hypoxia (5, 11, 25). Here, and in previous studies (5, 11), we demonstrated that a plurality of CRH neurons in the PVN project to the nTS. We also previously showed that most (∼90%) of the nTS projections that are activated by hypoxia are immunoreactive for CRH (5, 11).

### The Orexin-CRH-nTS Circuit Enhances the Peripheral Chemoreflex Mainly in the Active Phase of the Circadian Cycle

Current data demonstrate that silencing the CRH-nTS pathway blunts the strength of the peripheral chemoreflex solely in the active phase, similar to our previous study showing that Ox1R blockade with SB-334867 also reduces the strength of the reflex and to a similar extent as pathway blockade (11). In addition, CRH-nTS pathway inhibition in the active phase occluded the effect of Ox1R blockade on reflex strength. Together, our data suggest that in the active phase, orexin augments the reflex predominantly through nTS-projecting CRH neurons in the PVN, because if another pathway was involved, an effect of Ox1R blockade would persist following CRH-nTS silencing. Our previous findings support this concept. First, we showed that hypoxia activates PVN-projecting orexin neurons but not those projecting directly to the nTS (11). Second, knockdown of Ox1R in the PVN reduced the strength of the reflex and the activation (c-Fos immunoreactivity) of both the PVN and tyrosine hydroxylase-immunoreactive neurons in the nTS (11). Finally, we showed more specifically that OxR blockade reduced the hypoxia-induced activation of nTS-projecting CRH neurons (11). These and current observations are consistent with and support the concept that orexin neurons bolster the strength of the peripheral chemoreflex in the active phase through nTS-projecting CRH neurons.

Although chemogenetic activation of the CRH-nTS pathway had no effect on the strength of the reflex, we nevertheless found that Ox1R blockade reduced breathing in room air and its response to hypoxia. This suggests that orexin may be acting through Ox1R at a different location. However, this conclusion is not supported by our data from chemogenetic inhibition, which occluded the effect of Ox1R blockade. An alternative explanation is that the increase in CRH neuronal activity, through collateral innervation of the lateral and/or perifornical hypothalamus, increases orexinergic drive through the PVN, nTS, or other nuclei participating in the peripheral chemoreflex. There is recent evidence that supports the concept that CRH neurons facilitate the activation of orexin neurons (26). More research is needed to resolve the precise role of orexin on the activation of the PVN in response to hypoxia—especially on the role of CRH neurons and other PVN neuronal phenotypes projecting to the nTS.

We have previously demonstrated that orexin contributes to peripheral chemoreflex strength in both phases (13). Our current data suggest that Ox1R blockade also reduces the strength of the peripheral chemoreflex during the inactive phase, recapitulating our original findings with a dual OxR blocker (13). Although orexin neurons have higher activity in the active phase compared with the inactive phase, we have previously demonstrated that orexin neurons are indeed activated by hypoxia in the inactive phase (∼10%) (13). Thus, they have the potential to influence the reflex in this phase.

### Pathophysiological and Clinical Significance

The PVN is a central hub that mediates cardiorespiratory, neuroendocrine, and autonomic responses to multiple stressors. PVN activity is heightened in a variety of disease states including heart failure and hypertension; both of these disease states are associated with intermittent hypoxia and augmented sympathetic nerve activity (SNA). There is ample evidence that intermittent hypoxia elevates SNA via an increase in peripheral chemoreflex sensitivity (27, 28). All major neuronal phenotypes, including OT, AVP, and CRH neurons, have been shown to increase sympathetic activity through several pathways, including direct projections to preganglionic neurons in the intermediolateral cell column of the spinal cord and projections to the rostral VLM (3, 29). Hypoxia activates CRH neurons that project from PVN to the nTS (5, 11), but not those CRH neurons that project to the spinal cord (10). Intermittent hypoxia also activates CRH neurons in the medial parvocellular subnucleus of the PVN (18), but it was not clear whether these were neuroendocrine CRH neurons that projected to the median eminence to elicit HPA axis activation or, alternatively, were nTS-projecting. In the current study, we have shown that CRH neurons that project to the nTS contribute to the strength of the peripheral chemoreflex. The CRH-nTS pathway may therefore be involved in the elevated SNA and hypertension that is present in cardiovascular diseases associated with hypoxia.

## Data Availability

Data will be made available upon reasonable request.

## SUPPLEMENTAL MATERIAL

Supplemental Fig. S1: All supplemental material for this article is accessible at https://doi.org/10.6084/m9.figshare.30595343.
